# Prevalence and determinants of impetigo in Ghana: a cross-sectional study

**DOI:** 10.1186/s12879-024-09242-y

**Published:** 2024-03-26

**Authors:** Yaw Ampem Amoako, Dennis Odai Laryea, Abigail Agbanyo, Bernadette Agbavor, Michael Ntiamoah Oppong, Gloria Kyem, Kabiru Mohammed Abass, Renee van Bentum, Richard Odame Phillips, Ymkje Stienstra

**Affiliations:** 1 Kumasi Centre for Collaborative Research into Tropical Medicine, Kwame Nkrumah University of Science and Technology, Kumasi, Ghana; 2 School of Medicine and Dentistry, Kwame Nkrumah University of Science and Technology, Kumasi, Ghana; 3 Department of Internal Medicine/Infectious Diseases, University of Groningen, University Medical Center Groningen, Groningen, The Netherlands; 4 Komfo Anokye Teaching Hospital, Kumasi, Ghana; 5 Disease Surveillance Department, Ghana Health Service, Accra, Ghana; 6 Agogo Presbyterian Hospital, Agogo, Ghana; 7 Department of Clinical Sciences, Liverpool School of Tropical Medicine, Liverpool, UK

**Keywords:** Impetigo, Prevalence, Determinants, Scabies, Ghana

## Abstract

**Introduction:**

Skin diseases such as impetigo pose a significant public health challenge in low resource settings. Despite this, there is a dearth of epidemiological data on the prevalence of this condition in Ghana.

**Methods:**

We conducted a cross sectional study in three settings in Ghana: community members in East Mamprusi district in the North East region, a secondary school in Sekyere East district, and inmates of the Kumasi central prisons both in the Ashanti region. Following a period of training, we performed a standardised skin examination on each participant to assess for scabies and impetigo. We calculated the prevalence of each skin condition and investigated determinants of impetigo.

**Results/ findings:**

Of the 1327 participants [males 64.1% and median age 22 (16–29) years], 746 (56.2%) had scabies and 186 (14%) had impetigo which was usually very mild or mild in severity. Most participants with impetigo also had scabies (161/186, 86.6%). Having an itch [RR 6.05 (95% CI 2.53–14.47)], presence of scabies burrows [RR 1.99 (95% CI 1.54–2.59)], clinical scabies [RR 3.15 (2.11–4.72)] or being in preschool [RR 4.56 (1.78–11.67)] increased the risk for impetigo. A combination of the presence of clinical scabies, age, sex and itch most accurately predicted the odds of having impetigo.

**Conclusions:**

There is substantial burden of impetigo and scabies in Ghana. There is a need to institute measures to improve detection and control of these common dermatoses as part of Universal Health Coverage package to reduce the scourge of the diseases in this setting.

## Introduction

Skin diseases are one of the most common human diseases. They affect individuals of all ages and from all socio-economic backgrounds [1–3]. The detrimental effects of skin diseases on health include physical, psychologic and socio-economic impact on affected persons and their families [4–8].

Impetigo is a common bacterial skin infection that affects millions of people around the world, particularly children. In a previous systematic review [9], 162 million children globally were estimated to be affected by impetigo and those in resource-poor regions were most affected. A prevalence of 7% was reported among children in Africa compared to 29.7% and 15.5% in Oceania and Latin America respectively. Impetigo is primarily caused by the bacteria *Staphylococcus aureus* or *Streptococcus pyogenes*, and is highly contagious, making it a significant public health concern [9–11].

The pathogenesis of impetigo is complex and involves a combination of bacterial factors and host immune response. Crowded environments, such as schools and day care centres, increase transmission of impetigo in young children [12–14]. A serine protease inhibitor in scabies mites (SMSB4) interferes with the complement-mediated killing of *Staphylococcus aureus* by inhibiting the deposition of C3b, C4b and properdin on the bacteria surface thereby reducing opsonization, phagocytosis and further recruitment of neutrophils to the site of infection. Furthermore, complement inhibitors secreted by the mites, provide favourable conditions for the onset of *Staphylococcus aureus* co-infection in the scabies-infected microenvironment by suppressing the immediate host immune response [15]. These complement inhibitors secreted by scabies mites have also been reported to promote the growth of *Streptococcus pyogenes* [16] and this may account for the increased probability of culturing the bacteria from impetigo lesions when scabies is present [17]. Adults, particularly those who are immunocompromised (from medical conditions like malnutrition and diabetes mellitus) or have underlying skin conditions have increased susceptibility to impetigo [18, 19]. Additionally, skin trauma as occurs from scratching in response to pruritic conditions like scabies, eczema predispose to impetigo [20, 21].

Impetigo may affect skin anywhere on the body, but is most common around the nose and mouth, hands, and forearms, and in young children, the diaper area. The clinical presentation of impetigo can vary, depending on the causative agent and the severity of the infection. The most common presentation is the appearance of red sores or blisters, which can be itchy or painful. The lesions may be localized to a small area or may be more widespread and can spread rapidly to other parts of the body through direct contact or contact with contaminated objects. In addition, the formation of a yellowish crust over the affected area is a characteristic feature of impetigo. There are two forms of impetigo: non-bullous (crusted) and bullous (large blisters) with the non-bullous or crusted impetigo being most common (70% of cases). Occasionally, the lesions form ulcers (ecthyma) [22, 23]. Impetigo typically resolves within two to three weeks without scarring. Complications, though rare, may occur and range from cellulitis to septicemia and acute poststreptococcal glomerulonephritis [1, 21].

Treatment of impetigo typically involves the use of topical or systemic antibiotics, along with measures to promote wound healing and prevent further spread of infection. Topical antibiotics are more effective and preferable to oral antibiotics for limited impetigo. Systemic antibiotics are often reserved for more generalized or severe infections [22, 24].

Scabies, a pruritic skin infestation of the skin, is common in low resource settings and has been reported to increase the risk of impetigo [13, 20, 25]. In previous studies, individuals with scabies infestation were twice as likely to have active impetigo infection with 41.1% and 22% of participants with scabies cases also having impetigo in the Solomon Islands [25] and Ghana [20] respectively. Both scabies and impetigo are considered important dermatologic conditions and are common especially in low resource settings. This study aimed to establish the prevalence of impetigo among individuals with scabies in different regions and settings in Ghana, and to investigate the characteristics and epidemiologic associations/ determinants of impetigo.

## Methods

### Study setting

This cross-sectional study included individuals in 3 areas within Ghana: community members in East Mamprusi district in the North East region, a secondary school in Sekyere East district, and inmates of the Kumasi central prisons both in the Ashanti region of Ghana. We conducted skin examination of individuals in the 3 aforementioned areas of Ghana between October 2019 and December 2022 following reports of scabies outbreaks by health authorities. The study suffered delays due to the restriction on most public health activities in Ghana during the COVID-19 pandemic and so data collection in the prisons could only take place in December 2022.

The East Mamprusi district is largely rural with 68% of the population living in rural communities. Nearly half of the population are age 0–14 years, and the average household size is 8.6 persons per household. Most of the economically active population are engaged in agriculture for their employment. Most of the population reside in mud/mud bricks houses roofed with metal sheets or and thatch/palm leaf or raffia [26]. We published data of 283 participants from this area in an earlier study [20].

The Sekyere East district in the Ashanti region has 54.1% of the population residing in the urban areas compared to 45.9% in the rural areas. The district has an average household size of 4.5. Predominant occupations in the district are farming, forestry, and trading. Most people live in cement block/concrete houses with metal sheets as roofing material [27]. The Kumasi Central prison is a medium security facility located in Kumasi in the Ashanti region of Ghana; it had 1906 inmates (1886 males and 20 females) at the time of the study.

### Study design/ participant recruitment and study procedures

The methodology for patient selection from the scabies outbreak in East Mamprusi district have been previously published [20]. Briefly, in the East Mamprusi district, community members and students were invited for participation during a house-to-house and a school visit during a scabies outbreak investigation. In Sekyere East, students from the secondary/ technical school were included. The students were contacted in their classrooms where the study was explained after which they were invited to participate. With the students seated in rows in their classrooms, every third student seated on a row who provided informed consent was selected for enrolment. In the prisons, prisoners were contacted within their cells and informed about the assessment and consent sought. Every third prisoner within a cell who provided informed consent was enrolled. All examinations were performed within the infirmary of the prison.

The research team comprised medical doctors with clinical experience diagnosing scabies based on earlier activities in infectious diseases and/or public health. Additionally, a supplemental training program on the diagnosis of scabies, impetigo and other locally common skin conditions as well as the use of the IACS criteria for scabies diagnosis was provided to team members by the lead researcher (YAA).

Demographic and clinical details were recorded with the aid of a REDCap based questionnaire. Using the approach published previously [13], a standardized skin examination of exposed regions of the body: the feet and legs to the thighs, hands to the upper arms, neck, face and scalp was performed on all participants. Participants were required to have the designated body regions exposed prior to their skin examination. Scabies was diagnosed based on the criteria of the International Alliance for the Control of Scabies (IACS) [28]; the B1, B3, C1 and/or C2 (as shown in Table 1) were used for diagnosis in this study. Impetigo was diagnosed based on the presence of papules, pustules or ulcerative lesions with associated erythema, crusting or pus. The severity of scabies and impetigo were assessed based on the number of lesions present using previously published criteria [25]. For scabies, mild disease was 1 to 10 lesions present; moderate, 11 to 49 lesions; or severe, 50 or more lesions. Impetigo was categorised as: very mild, 1 to 5 lesions; mild, 6 to 10 lesions; moderate, 11 to 49 lesions; or severe, 50 or more lesions. Individuals with scabies and impetigo were treated per national guidelines [29]. Other skin diseases identified were either treated by the research team following national guidelines or referred to the health facility for appropriate care.

**Table 1:** Case definitions for scabies using the IACS criteria

Clinical category		Used in survey
Confirmed scabies		
A1 A2 A3	Mites, eggs or faeces on light microscopy of skin samples Mites, eggs or faeces visualised on individual using high powered imaging device Mite visualised on individual using dermoscopy	No No No
Clinical scabies		
*B1 B2 B3	Presence of burrows Typical lesions affecting male genitalia Typical lesions in a typical distribution and two history features (itch and contact history)	Yes No Yes
Suspected scabies		
C1 C2	Typical lesions in a typical distribution and one history feature (itch or contact history) Atypical lesions or atypical distribution and two history features (itch and close contact with an individual who has itch or typical scabies lesions in a typical distribution)	Yes Yes

*Burrows were not confirmed with dermoscopy in the study

### Data analysis

Descriptive statistics were used to summarize the study findings. Data in RedCap was exported into Microsoft Excel software version 2013 (Microsoft Corp., USA). Analysis was performed using STATA software version 14 (STATA Corp., USA) and SPSS statistical software (IBM SPSS statistics Version 20 (IBM Company, Armonk, NY, USA). Categorical data were analysed using the Chi-square test of association and the Fisher’s exact test where cell counts were below 5. To identify the set of variables that best predicted impetigo, we performed a binary logistic regression analysis with backward selection based on likelihood ratios. Sex, itch, and presence of clinical scabies were marked as categorical covariates. The variance of the model was assessed using Nagelkerke R squared and we determined the goodness-of-fit by the Hosmer-Lemeshow test.

## Results

There were 1327 participants included: 553 (41.7%) and 215 (16.2%) were from East Mamprusi and Sekyere East districts respectively and the remaining 559 (42.1%) were from the Kumasi central prisons. The majority were male (64.1%), and the median age of the participants was 22 (IQR 16–29) years. Twenty eight (28) participants were in preschool and all were from the East Mamprusi area.

### Prevalence of impetigo

One hundred and eighty-six participants (14%) had impetigo of varying severity: most had very mild (44.6%) or mild (43.5%) disease and none had severe disease. There was no significant difference in the age of participants with and without impetigo (Table 2). The median duration of itch in participants with impetigo was 30 (21–60) days. Furthermore, there was no difference in the duration of itch or rash in participants with and without impetigo.

Based on the IACS criteria, 56.2% of the 1327 participants were diagnosed with scabies. Scabies was mostly mild (56.2%) or moderate (35.6%) severity. Scabies lesions were mostly located on hands, fingers and finger webs. No cases of crusted scabies were observed. Most participants with impetigo had scabies (161/186, 86.6%) which was mostly of moderate severity (50%) (Table 2).

**Table 2:** Characteristics of participants with and without impetigo

Characteristic			Impetigo, n (%) = 186	No impetigo, n (%) = 1141	Total, n (%) = 1327	*p* value
Median age (IQR), years			20 (6–28)	22 (16–29)	22 (16–29)	0.61
Sex	Male		119 (64.0)	732 (64.2)	851 (64.1)	0.34
	Female		64 (34.4)	406 (35.6)	470 (35.4)	
	Missing		3 (1.6)	3 (0.2)	6 (0.4)	
Median (IQR) duration of itch, days			30 (21–60)	30 (21–90)	30 (14–60)	0.29
Median (IQR) duration of rash, days			30 (14–60)	30 (14–60)	30 (14–60)	0.79
Proportion with scabies			161 (86.6)	585 (51.3)	746 (56.2)	< 0.05
*IACS scabies category*		B1 (%)	92 (57.1)	272 (46.5)	364 (48.8)	< 0.05
		B3 (%)	52 (32.3)	293 (50.1)	345 (46.2)	
		C1 (%)	12 (7.5)	16 (2.7)	28 (3.8)	
		C2 (%)	5 (3.1)	4 (0.7)	9 (1.2)	
Positive contact history (%)			184 (98.9)	865 (75.8)	1049 (79.1)	0.001

Clinical scabies [RR 3.15 (95% CI 2.11–4.72)], having an itch [RR 6.05 (95% CI 2.53–14.47)], being in preschool [RR 4.56 (95% CI 1.78–11.67)], and presence of burrows [RR 1.99 (95% CI 1.54–2.59)] increased the risk of impetigo.

### Risk prediction model for impetigo

To identify the set of variables that best predicted impetigo, a regression analysis with backward selection based on likelihood ratios was performed. Variables included were age, sex, presence of clinical scabies, presence of scabies burrows, school status, prison status, rash, rash duration, itch, and itch duration. The most appropriate model predicting impetigo in the study population included the variables age, presence of clinical scabies, sex and itch (Nagelkerke R^2^ = 0.13, Hosmer-Lemeshow goodness-of-fit test *p* = 0.30). Itch and having clinical scabies produced a larger effect on the model (Table 3).

**Table 3:** Results of the binary logistic regression analysis on the variables best predicting impetigo in scabies participants

Variable	OR	95% CI
Clinical scabies		
No Yes	- 2.5	1.5–3.9
Age (per year)	0.96	0.95–0.98
Sex		
Female Male	- 0.9	0.6–1.3
Itch		
No Yes	- 4.3	1.7–11.4

OR: odds ratio; Nagelkerke R^2^ = 0.13; 95% CI: 95% confidence interval

### Treatment received

83% (83%, 154/186) of participants with impetigo had not received any treatment for the condition. Only thirteen (10 mild and 3 moderate) with impetigo had received treatment for their condition and this was with oral amoxicillin (3 persons) or topical cream (10 persons). Further, none of the participants with very mild impetigo (< 5 lesions) had received any treatment for the condition. Only 18% (134/746) of participants with scabies had already received treatment for their scabies prior to the assessment.

## Discussion

This study found an overall impetigo prevalence of 14% in 1327 participants evaluated in Ghana. Impetigo was mostly very mild or mild in severity. Scabies and impetigo commonly occurred together, and scabies was found to strongly predispose to the development of impetigo. Being in preschool, having an itch, burrows and clinical scabies increased the risk of impetigo. Further, majority of individuals with impetigo had not received any treatment for their condition at the time of assessment. In a survey conducted over 4 decades ago in rural Ghana [30], a slightly higher impetigo prevalence of 19.4% was found. Acheampong et al. [31] reported a prevalence of 11.7% among individuals with scabies in a rural community in the Ashanti region of Ghana with peak rates occurring in the 5–9-year-old age group. In the current study, 88% of those with active impetigo had very mild or mild disease; this is similar to a previous study of rural communities in the Solomon Islands [25] where 90% of participants had very mild or mild disease. Romani and colleagues reported an impetigo prevalence of 23.4% in their study of island communities in Fiji where the prevalence of scabies was 36.4% [32], a figure which is less than the 56.2% found in the current study. Yet, higher rates of impetigo have been reported in the Solomon Islands (43%) and among Aboriginal communities in Australia (49%) [21]. Impetigo has been reported to affect about 140,495,000 individuals globally and features in the top 50 causes of disease in humans [1]. This has necessitated calls to include its prevention and treatment as part of general skin health assessment among at risk populations. It is important to recognise this significant burden of disease in Ghana in order to develop and implement context specific strategies to address it.

We found that presence of clinical scabies, and itch were predictors of impetigo suggesting that scabies infestation is an important risk factor for bacterial infection of the skin. This association is consistent with previous reports from Fiji [33] and northern Ghana [20]. Furthermore, age and sex were predictive of impetigo. We found that the risk of impetigo was high among preschool children. In Ghana, education at the preschool level is associated with large class sizes and overcrowding [14]. Further, preschool children are less likely to adhere to physical distancing and hand hygiene measures; situations that can facilitate ongoing spread of impetigo and scabies via direct contact. There is the need to further enhance strategies that promote the early detection and knowledge of how to further stop the transmission of impetigo including the use of water and soap as part of control measures for skin NTDs in endemic communities [34]. In a survey conducted among school children in Samoa [35], the prevalence of impetigo was 57.1% and associations between active impetigo and age and sex were noted, with younger children and males more commonly affected (aOR 2.8 [1.8–4.7] and aOR 1.8 [1.3–2.5] respectively). Although both Ghana and Samoa are countries with tropical weather conditions, the differing characteristics of the study participants might account for the observed differences in the studies.

In low resource settings, scabies and impetigo contribute to a significant burden of disease for children and adults. Scabies infection can predispose to secondary bacterial infection with group A Streptococci (GAS) and *Staphylococcus aureus*. GAS are nephritogenic and can lead to glomerulonephritis, further increasing the socio-economic burden on the individual and the health system [1, 36]. In the current study, 56.2% of participants had scabies and 87% of individuals with impetigo also had scabies. We found that scabies is a strong driver for impetigo infection (RR 3.15, *p* < 0.05) and this risk was high in individuals with moderate and severe scabies; this agrees with previous reports of scabies increasing the risk of developing impetigo in affected individuals [20, 25, 32]. There is a need for coordinated strategies to help address the scourge of these twin communicable diseases within resource limited settings like Ghana. Most individuals with impetigo in our study had not been diagnosed or received appropriate treatment. This may indicate a low awareness of the condition among the population. Further, it may also reflect an under recognition and under treatment even among health workers who attend to vulnerable populations as those studied. Earlier and quicker access to care for scabies may be useful to reduce the impact of scabies and consequently impetigo on affected individuals. Furthermore, there is an urgent need to enhance education on scabies and impetigo and implement integrated case search programmes to facilitate early detection and to align with the global objective of the World Health Organization (WHO) Road Map for 2021–2030 for the control, elimination, and eradication of Neglected Tropical Diseases (NTDs) [37, 38].

In nonendemic settings, topical agents, such as ozenoxacin, retapamulin, and minocycline have been investigated for the treatment of impetigo while in endemic settings, topical and systemic antibiotics have been used for treatment of affected individuals [39]. Several trials conducted in endemic settings have shown mass drug administration (MDA) intervention as a promising public health strategy for the control of scabies and impetigo [40–42]. Thean et al. [11] conducted a prospective study on the impact of scabies on hospital admissions for skin and soft tissue infections (SSTIs) such as impetigo, abscess, cellulitis, pyomyositis and necrotizing fasciitis in Fiji and reported that scabies and impetigo were substantial contributors to hospital admissions for SSTIs especially among the young and elderly population.

Ivermectin based MDA has been found to result in a 94% relative reduction in the prevalence of scabies and a 67% relative reduction in impetigo 12 months after administration. Further benefit on prevalence of scabies and impetigo was observed even after 24 months [41]. Another study [42] reported a sustained impact of a single round of ivermectin and azithromycin MDA on scabies and impetigo prevalence 3 years after the intervention.

The addition of azithromycin to an ivermectin-based MDA had no apparent benefit in further reducing the rates of impetigo, emphasizing the fact that the reduction in impetigo prevalence was largely related to the decrease in scabies. Rather, there was an increase in macrolide-resistant *Staphylococcus aureus* strains after adding azithromycin to an MDA regimen for trachoma [43] and highlights the need to carefully assess the use of antibiotics in such programmes.

Among individuals with impetigo, antibiotics may be given to reduce the risk of contagious spread within the affected individual and to others, quicken resolution of lesions, and reduce the risk of complications in vulnerable populations. However, non-bullous impetigo in some individuals resolves spontaneously by about 7 days [44]; this implies antibiotics are not always needed. Informed decision making on antibiotic use in impetigo should balance the benefits of use against the potential risk of development of antibiotic resistance within the context of good antimicrobial stewardship programmes.

The first line treatments (25% Benzyl benzoate lotion for scabies and topical mupirocin or flucloxacillin or Amoxicillin/ clavulanate for impetigo) [29] are covered under the National Health Insurance scheme (NHIS) in Ghana. However, individuals in the vulnerable population are usually in the low socioeconomic class and may not even be enrolled on the insurance scheme and may have to make out-of-pocket payments for health care. Furthermore, insured clients of Ghana’s NHIS seeking health care in accredited health facilities located in poor regions of the country have been reported to make out-of-pocket payments for drugs that are covered by the scheme [45]. The out-of-pocket payments are largely attributed to unavailability of drugs at the health facilities thus necessitating a need for affected individuals to get their medications elsewhere. Such costs limit access to care for these vulnerable individuals who are most at risk for scabies and impetigo. A high reliance on out-of-pocket payments can impede progress towards achieving the Sustainable Development Goals on Universal Health Coverage (SDG 3.8) and ending the epidemic of neglected tropical diseases (SDG 3.3) and lead to health inequalities. Scabies treatment is further hampered by a need to treat household and other contacts. Given the high prevalence of scabies in this population, mass drug administration (MDA) [40] will be an optimal control strategy for addressing the high disease burden. Additional control measures will include community education, and implementation of water, sanitation and hygiene (WASH) programmes within affected populations. Screening for impetigo should be included in the integrated care strategy for skin diseases in Ghana.

### Limitations

While this is the first dedicated study on impetigo in Ghana since a previous report by Belcher et al. in 1977 [30] and contributes important new information on the subject, there are a number of limitations. The study population had an under-representation of younger children and an over-representation of older people, possibly due to sampling of a senior secondary school and a prison population. However, these institutionalised populations are known to have a high risk for scabies and associated impetigo infection. The findings show a substantial burden of scabies and impetigo among the study population. Yet, the study was conducted in communities following reports of scabies outbreaks by health authorities and it is probable the prevalence of scabies and impetigo might have been over-estimated. Although diagnosis of scabies was entirely clinical and did not include dermoscopy or scraping of scabies mites, we used the IACS criteria which has been shown to be useful for scabies diagnosis by mid-level health workers and non-experts in field conditions [20, 46]. Further, we did not conduct any bacteriology tests to confirm the presence of bacterial infection of impetigo lesions. Yet the diagnosis of scabies and impetigo was performed by health workers with experience in infectious diseases and public health who received additional training in relevant methods, prior to the conduct of the assessment.

## Conclusion

This study describes a considerable burden of disease attributed to impetigo and scabies in Ghana. Scabies infestation was strongly associated with an increased risk of impetigo. These findings provide baseline data on the epidemiology of impetigo and will be valuable in addressing the public health challenge posed by the condition. There is a need to increase awareness of impetigo and increase public health efforts to address the scourge of the disease in Ghana.

## Data Availability

All data generated or analysed during this study are included in this published article.
